# Living donor liver transplantation for advanced hepatocellular carcinoma including macrovascular invasion

**DOI:** 10.1007/s00432-021-03665-9

**Published:** 2021-06-12

**Authors:** Abu Bakar Hafeez Bhatti, Wajih Naqvi, Nusrat Yar Khan, Haseeb Haider Zia, Faisal Saud Dar, Zahid Amin Khan, Atif Rana

**Affiliations:** 1 Division of Hepato-Pancreato-Biliary Surgery and Liver Transplantation, Shifa International Hospital Islamabad, Sector H-8/4, Pitras Bukhari Road, Islamabad, 44000 Pakistan; 2 Department of Surgery, Shifa Tameer-e-Millat University Islamabad, Islamabad, Pakistan; 3 Division of Radiology, Shifa International Hospital Islamabad, Islamabad, Pakistan

**Keywords:** Alpha fetoprotein, Portal vein tumor thrombus, Locoregional therapy, Recurrence, Overall survival

## Abstract

**Background:**

The indications for liver transplantation (LT) in patients with hepatocellular carcinoma (HCC) continue to evolve. The aim of this study was to report outcomes in patients who underwent living donor liver transplantation (LDLT) for HCC outside traditional criteria including macrovascular invasion (MVI).

**Methods:**

We reviewed outcomes in patients who met the University of California San Francisco (UCSF) criteria (*n* = 159) and our center-specific criteria (UCSF+) (largest tumor diameter ≤ 10 cm, any tumor number, AFP ≤ 1000 ng/ml) (*n* = 58). We also assessed outcomes in patients with MVI (*n* = 27).

**Results:**

The median follow was 28 (10.6–42.7) months. The 5 year overall survival and risk of recurrence (RR) in the UCSF and UCSF + group was 71% vs 69% (*P* = 0.7) and 13% vs 36% (*P* = 0.1) respectively. When patients with AFP > 600 ng/ml were excluded from the UCSF + group, RR was 27% (*P* = 0.3). Among patients with MVI who had downstaging (DS), 4/5(80%) in low-risk group (good response and AFP ≤ 100 ng/ml) and 2/10 (20%) in the high-risk group (poor response or AFP > 100 ng/ml) were alive at the last follow-up. When DS was not feasible, 3/3 (100%) in the low-risk group (AFP ≤ 100 ng/ml + Vp1-2 MVI) and 1/9 (9.1%) in the high-risk group (AFP > 100 or Vp3 MVI) were alive. The 5 year OS in the low-risk MVI group was 85% (*P* = 0.003).

**Conclusion:**

With inclusion of AFP, response to downstaging and degree of MVI, acceptable survival can be achieved with LDLT for HCC outside traditional criteria.

**Supplementary Information:**

The online version contains supplementary material available at 10.1007/s00432-021-03665-9.

## Introduction

Liver transplantation (LT) is the most effective treatment for patients with cirrhosis and small hepatocellular carcinoma (HCC) (Costentin et al. 2019). Despite being restrictive, Milan criteria (single tumor ≤ 5 cm, upto 3 tumors ≤ 3 cm) remain the benchmark for LT in HCC. LT for tumors within Milan criteria is associated with low recurrence rate and a 5 year survival of 60–80% (Mazzaferro et al. 1996; Toso et al. 2011). The University of California San Francisco criteria (UCSF) (Single tumor ≤ 6.5 cm, up to 3 tumors ≤ 4.5 cm, total tumor diameter ≤ 8 cm), was proposed in the year 2001, and has outcomes comparable to Milan criteria (Yao et al. 2001, 2007). A number of other expanded criteria have been proposed to further increase transplant eligibility (Duvoux et al. 2012; Mazzaferro et al. 2018; Sapisochin et al. 2016; Lei et al. 2014). Living donor liver transplantation (LDLT) is a viable alternative to deceased donor liver transplantation (DDLT) for HCC. Worldwide, LDLT centers have expanded the cutoffs on tumor size and number to increase transplant eligible patients (Hong et al. 2016; Lee et al. 2016, 2008). While most of the expanded criteria in DDLT and LDLT allow modest expansion in transplant pool, the more liberal criteria often mandate a preoperative biopsy or PET scan for patient selection (Sapisochin et al. 2016; Lei et al. 2014; Lee et al. 2016). Although prognostically significant, widespread adoption of preoperative biopsy has been limited by presumed risks of tumor seeding, bleeding, and misdiagnosis (Sparchez et al. 2018). The National Cancer Center Korea (NCCK) criteria used PET scan to select patients with HCC ≤ 10 cm irrespective of tumor number (Lee et al. 2016). Factors such as low sensitivity, cost effectiveness, and variable SUV cutoffs limit wide spread utility of PET scans for selecting HCC patients for transplantation (Lu et al. 2019).

At our center, LDLT has been routinely performed since 2012. We developed our own center-specific selection criteria for LDLT in patients with HCC. Since 2012, patients within UCSF criteria and those outside UCSF criteria (maximum tumor size ≤ 10 cm, any tumor number) with AFP < 1000 ng/ml were offered LDLT. In addition, LDLT was also offered to selected patients with macrovascular invasion (MVI).

The objective of this study was to share our experience with LDLT for HCC using expanded transplant criteria.

## Materials and methods

Between April 2012 and September 2019, 874 patients underwent LDLT at our center. Among them, 244 patients with a preoperative diagnosis of HCC, were reviewed retrospectively.

Our patient and donor selection criteria and workup have been described elsewhere (Dar et al. 2015, 2018). The decision to proceed with LDLT was finalized in multi-disciplinary team meeting and liver transplant listing meeting. These meetings include team members from transplant surgery, hepatology, radiology, anesthesiology, and administrative committee. In addition, all potential donors were assessed independently by donor advocates.

### Patient selection for LDLT

The radiological diagnosis of HCC was confirmed on dynamic imaging (CT scan or MRI) with an arterially enhancing lesion ≥ 1 cm demonstrating wash out on venous phase (Bruix and Sherman 2011). LDLT was only considered if there was no evidence of extra hepatic metastases and main portal vein tumor thrombosis (PVTT). All patients within UCSF criteria were offered upfront LDLT. In addition patients outside UCSF criteria (largest tumor size upto 10 cm, any tumor number and AFP < 1000 ng/ml) (UCSF +) were also considered for upfront LDLT. Patients with AFP > 1000 ng/ml and UCSF + tumors were considered for downstaging (DS) [(Transarterial chemo embolization (TACE), radio frequency ablation (RFA), microwave ablation (MWA), percutaneous ethanol ablation (PEA)]. In patients with an anticipated delay of > 3 months, locoregional therapy (LRT) was used as bridging therapy. For downstaging, TACE was performed routinely, while in selected patients (e.g. multi focal bilobar disease), it was combined with other ablative therapies. A drop in AFP to < 1000 ng/ml and radiological response based on modified response evaluation criteria in solid tumors (mRECIST) at 6–8 weeks was used to determine effectiveness of LRT and candidacy for LDLT (Lencioni et al. 2010). For bridging, RFA, MWA. PEA or TACE was considered based on the size and location of the tumors and underlying liver failure. In the post-transplant period, an annual surveillance CT scan was performed for the first two years with six monthly AFP levels. After two years, AFP and US was performed at 6 months interval. Sorafenib was used in patients with high-risk HCC (MVI, poor grade, tumors outside UCSF criteria) one month after LT. High-risk patients had their first surveillance CT scan 3 months after LT.

### LDLT for macrovascular invasion

During the study period, patients with HCC and macrovascular invasion (MVI) also underwent LDLT. We reviewed the pre-transplant imaging and patients who fulfilled the A-VENA criteria for MVI were included (Sherman et al. 2019). With regards to PVTT, patients with tumor thrombosis in segmental branches (Vp1-2), and lobar branches (Vp3) were considered for LDLT (Kanehara 2003). All these patients were considered for downstaging with response evaluation and an observation period (4–6 months). Only those with stable disease (no interval progression to extra hepatic disease and substantial rise in AFP) were offered LDLT. In patients with decompensated liver disease precluding DS, LDLT was offered selectively. All these patients had informed discussions regarding the higher risk of post-transplant recurrence. This was subsequently documented in the patient files by the transplants surgeons and hepatologists.

### Study design and statistical analysis

For this study, we compared demographics, etiology, tumor-related features, MELD score, and AFP for UCSF and UCSF + groups. Frequencies with percentage were reported for categorical data while medians with inter quartile range (IQR) were reported for interval data. For categorical variables, chi-squared and Fischer test were used while Mann–Whitney *U* test was used for interval variables. Overall survival (OS) was calculated by subtracting date of death from the date of transplantation. For survival analysis, Kaplan–Meier curves were used and Log rank test was used to determine significance. We also looked at the impact of AFP > 600 ng/ml on survival since it has been shown to be a strong predictor of outcomes (Bhatti et al. 2020a, b). On receiver operator curves (ROC) analysis, An AFP cutoff of 600 ng/ml (AUC = 0.77, *P* < 0.001) was a significant factor for recurrence. Among patients with MVI, we developed prognostic groups based on (1) response to DS, (2) AFP < or > 100 ng/ml, (3) Vp1-2 versus Vp3 PVTT as shown previously by other groups (Choi et al. 2017; Soin et al. 2020). Patients with complete or partial response were categorized as good responders while those with stable or progressive disease were considered as poor responders to LRT. A *P* value < 0.05 was considered statistically significant. The institutional review board and hospital ethics committee approved the study (IRB #433-1253-2020).

## Results

The median follow-up from transplantation was 28 (10.6–42.7) months. Median AFP was 16.2 (5.3–81) (range = 0.7–5129) ng/ml. The 5 year OS for the entire cohort was 71%. The 5 year OS with an AFP < or > 600 ng/ml was 74% and 27% (P = 0.01) (not shown). Table 1 shows the operative details in our cohort. There was no donor mortality.

**Table 1:** Surgical details in patients who underwent LDLT for HCC

Operative details	LDLT for HCC (*n* = 244)
Graft to recipient weight ratio, median(IQR)	0.99 (0.85–1.1)
Cold ischemia time, median (IQR) (min)	40 (24–58)
Warm ischemia time, median (IQR) (min)	37 (30–45)
Duration of surgery, median (IQR) (h)	8.3 (8–9.3)
> One hepatic vein reconstruction, number (%)	164 (67.2)
> One bile duct reconstruction, number (%)	69 (28.2)
Blood loss, median(IQR) (ml)	1500 (875–2400)
ICU stay, median (IQR) (days)	4 (4–6)
Hospital stay, median (IQR) (days)	15 (13–18)

### *UCSF and UCSF* + *groups*

We looked at various patient and tumor-related features in the UCSF (*n* = 159) and UCSF+ (*n* = 58) group (Table 2). Other than tumor size and number, there was a significant difference in median AFP level (*P* = 0.01) and microvascular invasion (*P* = 0.001). There was no significant difference with regards to HCC with AFP > 600 ng/ml and poor differentiation.

**Table 2:** Patient characteristics and tumor factors in patients within and outside UCSF criteria

	UCSF HCC (*n* = 159)	UCSF + HCC (*n* = 58)	*P* value
Mean age, SD (years)	53.2 ± 7.4	51.6 ± 6.4	0.1
Males, *n* (%)	133 (83.6)	45 (77.6)	0.3
HCV infection, *n* (%)	119 (74.8)	41 (70.7)	0.5
HBV infection, *n* (%)	26 (16.4)	11 (19)	0.6
MELD score, median (IQR)	19 (14–24)	16.5 (12–22)	0.06
AFP at the time of transplant (ng/ml), median (IQR)	10.6 (4.8–54.5)	32.4 (9–86.2)	0.01
AFP > 600 (ng/ml), *n* (%)	11 (6.9)	4 (6.9)	1
Tumor size on imaging, median (IQR)	2.3 (1.7–3.4)	4.5 (2.9–6.3)	< 0.001
Tumor number on imaging, median (IQR)	1 (1–2)	4 (3–6)	< 0.001
Pre-op locoregional therapy, *n* (%)	32 (20.1)	14 (24.1)	0.5
Tumor size on explant (cm), median (IQR)	2.5 (1–3.7)	5 (3–6)	< 0.001
Tumor number on explant, median (IQR)	1 (1–2)	3 (2–5)	< 0.001
Poor grade, *n* (%)	47 (29.5)	16 (27.6)	0.2
Microvascular invasion, *n* (%)	41 (25.8)	29 (50)	0.001

### Outcomes with LDLT

**Fig. 1:**
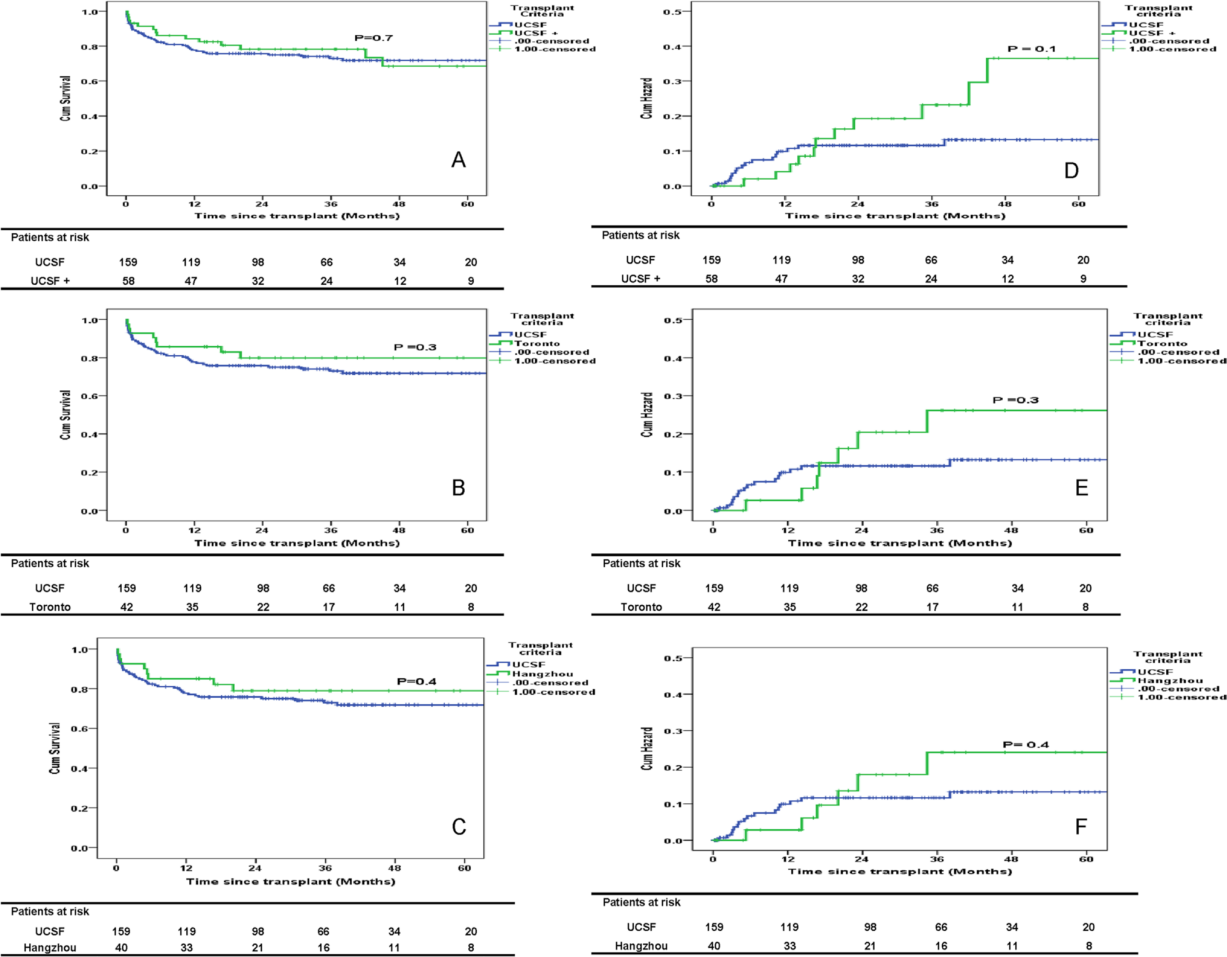
**A**–**C** Overall survival in patients fulfilling UCSF, UCSF+, Toronto and Hangzhou criteria. **D**–**F** Recurrence risk in patients fulfilling UCSF, UCSF+, Toronto and Hangzhou criteria

**Fig. 2:**
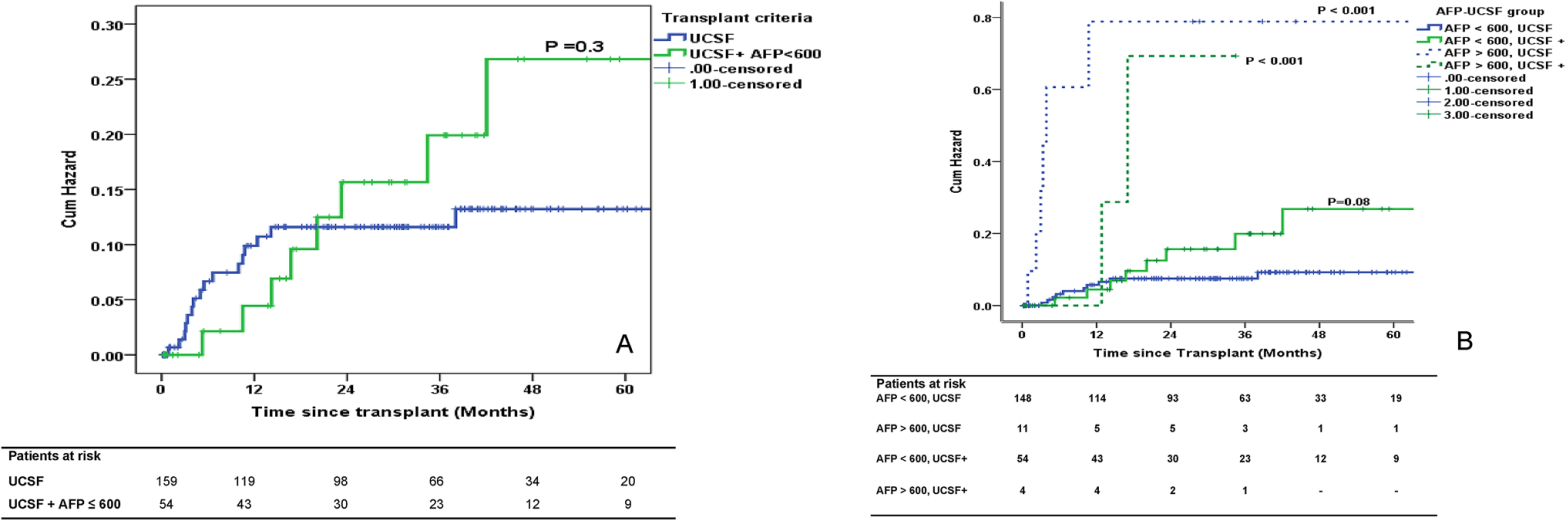
**A** Risk of recurrence in the UCSF group (*n* = 159) and UCSF+ AFP < 600 ng/ml group (*n* = 54). **B** Risk of recurrence risk with AFP < or > 600 ng/ml and UCSF and UCSF+ group

The actual survival rate in the UCSF and UCSF+ group was 117/159 (73.5%) and 44/58 (75.8%) (*P* = 0.7). The estimated 5 year OS was 72% and 69% for UCSF and UCSF+ group (*P* = 0.7) (Fig. 1A). The 5 year OS was 79% for patients who met Toronto criteria and Hangzhou criteria, and was comparable to patients within UCSF criteria (Fig. 1B, C). Among patients in the UCSF+ group, 16/58 (27.5%) would have been excluded under Toronto criteria and 18/58 (31%) under Hangzhou criteria. The recurrence risk (RR) for the UCSF and UCSF+ group was 13 and 36% (*P* = 0.1) (Fig. 1D). The RR was 26% and 24% for patients who met Toronto and Hangzhou criteria (Fig. 1E, F). When patients with AFP > 600 ng/ml (*n* = 4) were excluded from the UCSF+ group, RR was 27% (*P* = 0.3) (Fig. 2a). The RR was > 50% in patients with AFP > 600 ng/ml both in UCSF and UCSF + groups (Fig. 2b).

### LDLT for macrovascular invasion

**Fig. 3:**
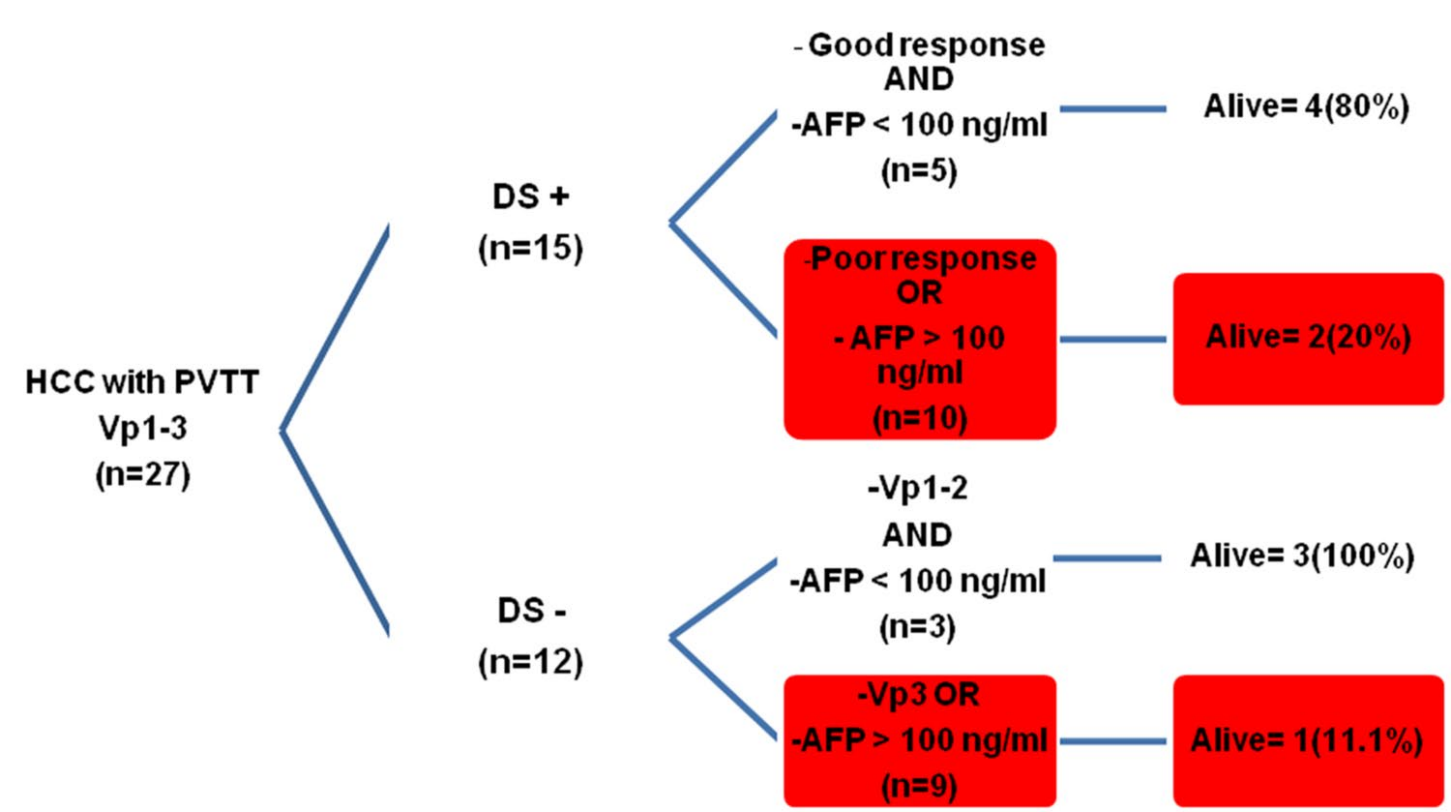
LDLT for HCC and macrovascular invasion (*n* = 27), the red boxes represent unfavorable groups

**Fig. 4:**
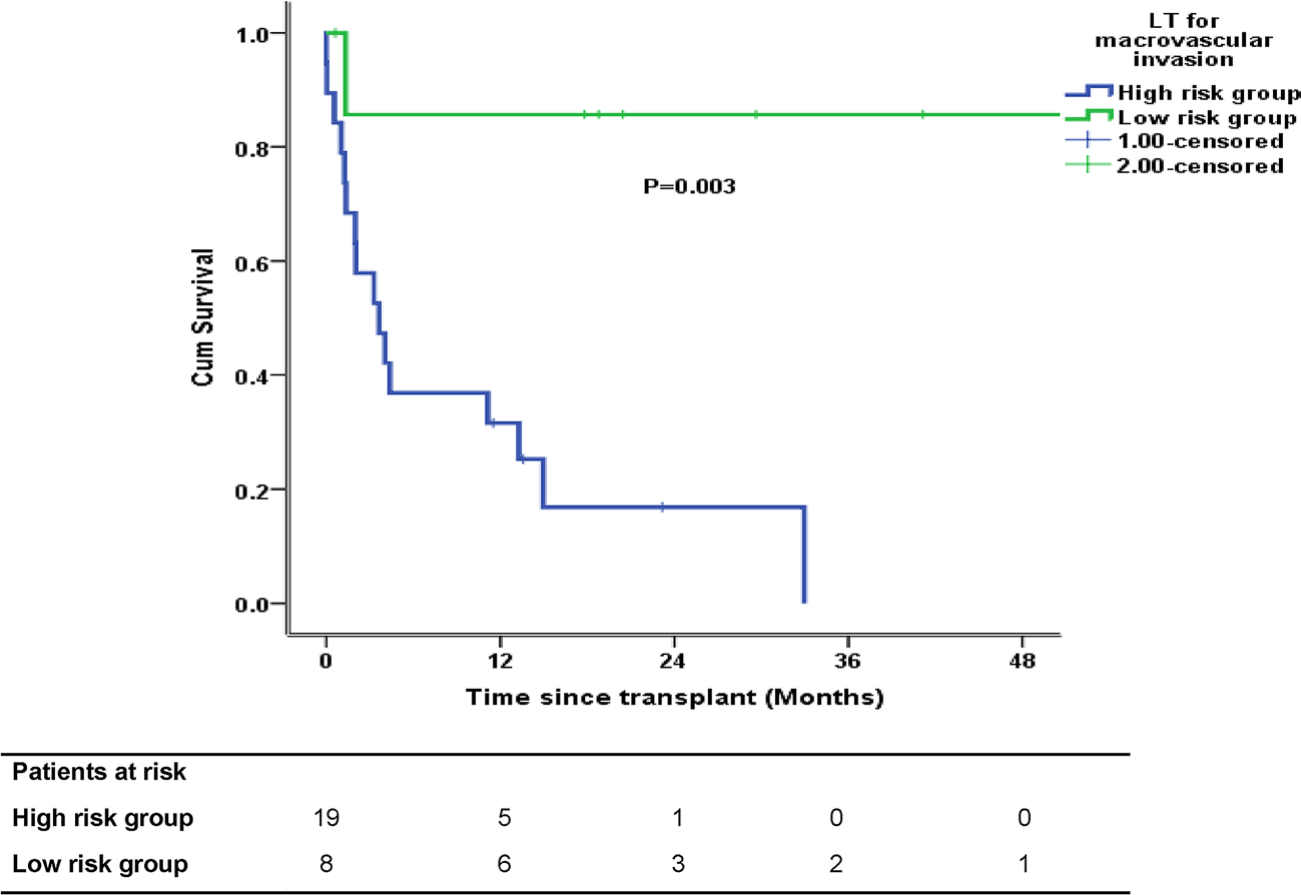
The estimated 5 year of overall survival in low- (*n* = 8) and high-risk (*n* = 19) macrovascular invasion groups

Median AFP in patients with MVI was 155 (16–702) ng/ml and AFP was < 100 ng/ml in 11 (40.8%) patients as shown in Table 3. The actual number of deaths in patients with an AFP ≤ or > 100 ng/ml was 4 (36.3%) and 13 (86.7%) (*P* = 0.04), respectively. On explant, the median largest tumor diameter was 5.5 (2.5–6) cm and tumor number was 2 (1–4). Figure 3 shows the survival in patients with MVI based on various prognostic groups. The estimated 5 year OS was 85% in the low-risk group and was not reached in the high-risk group (*P* = 0.003) (Fig. 4).

**Table 3:** Tumor-related features in patients with macrovascular invasion who underwent living donor liver transplantation

Factors	HCC-MVI (*n* = 27)	Death during follow-up	*P* value
Type of tumor thrombus, *n* (%)			
Vp1-2	16 (59.2)	8 (50)	0.1
Vp3	11 (40.8)	9 (81.8)	
Downstaging, *n* (%)			
Yes	15 (55.6)	9 (60)	> 0.99
No	12 (44.4)	8 (66.7)	
AFP at transplant (ng/ml), *n* (%)			
< 100	11 (40.8)	4 (36.3)	0.04
> 100	16 (59.2)	13 (81.2)	
Downstaging, *n* (%) (*n* = 15)			
Responders	9 (33.4)	4 (44.5)	0.2
Non-responders	6 (22.3)	5 (83.3)	
Waiting period, *n* (%)			
4–6 months	17 (63)	11 (64.7)	> 0.99
< 4 months	10 (37)	6 (60)	
Tumor differentiation			
Well-moderate	18 (66.7)	12 (66.7)	0.6
Poor	9 (33.3)	5 (55.6)	
Microvascular invasion			
Not seen	11 (40.7)	6 (54.5)	0.4
Seen	16 (59.2)	11 (68.7)	
Pathological complete response to downstaging (*n* = 15)			
Yes	2 (13.3)	1 (50)	> 0.99
No	13 (86.7)	5 (38.4)

### Management of recurrence

There were 27/217 (12.5%) recurrences in patients with HCC without MVI (supplementary table). Out of these, two patients had solitary hepatic recurrence. One of these patients was managed with TACE and sorafenib. The other patient underwent MWA and later required surgical excision of an isolated diaphragmatic recurrence three years after transplant. All other patients had recurrence involving multiple sites and were offered palliation with sorafenib. Three patients with painful bone metastases received palliative radiation. Table 3 shows the site of metastases in patients who underwent LDLT for HCC.

There were 5/27 (18.5%) recurrences in patients with HCC and MVI. All patients had recurrence involving multiple sites. One patient received palliative radiation while sorafenib was considered in all patients.

## Discussion

A number of expanded transplant criteria have been proposed for HCC in the DDLT and LDLT setting (Duvoux et al. 2012; Mazzaferro et al. 2018; Sapisochin et al. 2016, Lee et al. 2008, Choi et al. 2017). Most of these lead to a modest expansion in transplant pool. A pre-transplant biopsy appears to be a pre-requisite for more liberal expansion on tumor size and number and excludes patients with aggressive biology (Sapisochin et al. 2016; Lei et al. 2014). Although with this approach, survival comparable to Milan and UCSF criteria might be achieved, the use of pre-transplant biopsy remains debatable in clinical practice. We used AFP < 1000 ng/ml instead of biopsy, in patients outside UCSF criteria for patient selection. AFP > 1000 ng/ml is associated with increased risk of post-transplant recurrence even with HCC fulfilling UCSF criteria and recently has been incorporated into United Network for Organ Sharing (UNOS) HCC staging for patient listing (DuBay et al. 2011; Hameed et al. 2014; Bhatti et al. 2020). The OS achieved with tumor size cutoff of 10 cm and AFP < 1000 ng/ml was comparable to UCSF, Toronto, and Hangzhou criteria. Consistent with our previous experience as well as that from other centers, AFP > 600 ng/ml was a poor prognostic factor for patients in the UCSF and UCSF+ group with > 50% RR (Wong et al. 2019; Bhatti et al. 2020). Although, RR was not significantly different in the UCSF and UCSF+ groups, it is likely that statistical significance was not reached due to relatively lower patient number in the UCSF+ group. Nevertheless, 5 year RR of 36% in the UCSF+ group is acceptable and could be further reduced with an AFP cutoff of 600 ng/ml. Late recurrences were more common in the UCSF+ group and might have contributed to similar OS in the two groups.

Tumor size > 10 cm is associated with high risk of MVI, poor differentiation, and metastases (Wu et al. 2018). In the context of LDLT, tumor size cutoff of 10 cm has been used in combination with PET scan for patient selection by NCCK (Lee et al. 2016). The sensitivity of PET scan for HCC varies with tumor grade and location of metastases and optimal SUV cutoffs to predict recurrence are yet to be established. Moreover, it needs technical skill and experience for interpretation, and remains a costly investigation (Lu et al. 2019). Nevertheless, PET scan has become an invaluable clinical investigation in the pre-transplant workup of HCC patients. The clinical utility of pre-transplant AFP in patient selection for transplantation is already well established (Wu et al. 2018; Halazun et al. 2017). AFP is easily available, reproducible, and cost-effective investigation. The dynamic nature of AFP enables effective decision-making in patients receiving various locoregional treatments in the pre-transplant setting (Bhatti et al. 2020). In patients with advanced HCC, AFP in combination with other biomarkers like AFP L-3 and PIVKAII might improve patient selection for curative treatments. An AFP L-3 < 35% and PIVKAII < 400 mAU/ml allows safe expansion on tumor size and number while HCC exceeding these cutoff might yield unacceptable results even in patients fulfilling traditional criteria (Chaiteerakij et al. 2015; Kaido et al. 2013).

**Fig. 5:**
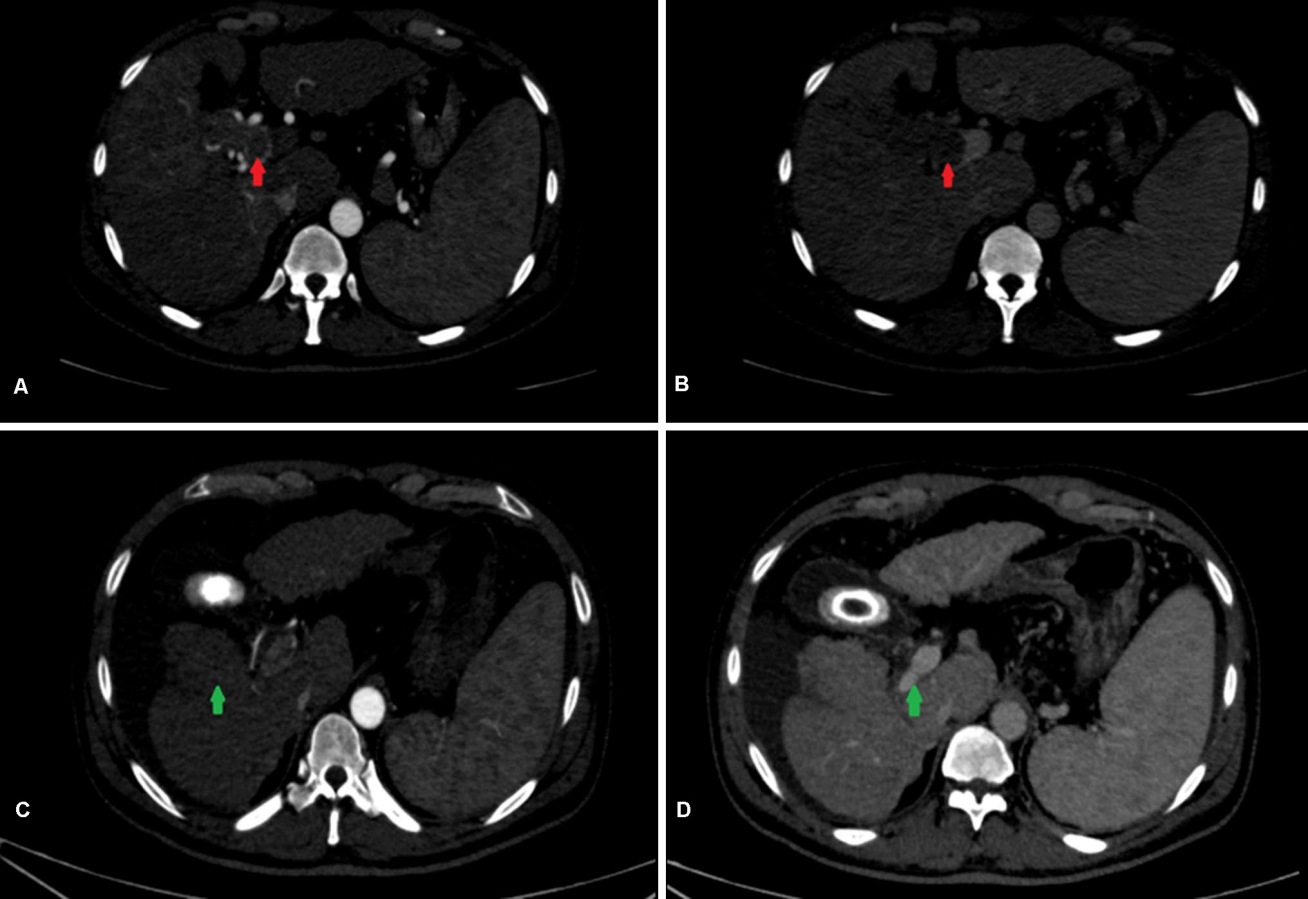
Long-term survival (60 months) after LDLT with prior downstaging for macrovascular invasion + HCC. **A** and **B** Liver dynamic CT scan in a patient with multi focal HCC, largest tumor size 10 cm and right portal vein tumor thrombus (red arrows). **C** Post TACE × 4 scan 12 months later, showed significant response to treatment in segment 5 (green arrow) and reduction in portal vein expansion. **D** Re canalization of right portal vein with significant resolution of portal vein tumor thrombus (green arrow), histopathology confirmed a poorly differentiated necrotic tumor with foci of residual HCC

Traditionally, MVI has been a relative contraindication to LDLT. However, in carefully selected patients with other positive prognostic features, LT can achieve acceptable long-term survival. Some of these factors include low AFP (10–100 ng/ml), good response to DS and Vp1-2 MVI (Assalino et al. 2020; Bhatti et al. 2020; Mehta et al. 2017; Lee et al. 2017). Our results are consistent with these reports and patient with favorable prognostic factors had acceptable outcomes. At our center, all patients with MVI undergo DS if feasible with an observation period of 4–6 months. We have shown that acceptable post-transplant survival is possible in a small group of carefully selected patients with Vp3 PVTT (Fig. 5). These patients have good response to DS based on mRECIST and an AFP < 100 ng/ml at the time of transplant. Patients with partial response, stable or progressive disease do poorly after LDLT. When DS is not feasible, patients with Vp1-2 MVI can be considered for upfront LDLT provided AFP at the time of transplant is < 100 ng/ml. Otherwise, outcome remains dismal and LT should be discouraged in patients with MVI.

Our findings have prompted certain modifications in our protocol for LDLT in HCC. We routinely check PIVKAII levels in all patients with HCC. Patients with AFP > 1000 ng/ml, both in UCSF and UCSF+ groups undergo downstaging. All patients with MVI are evaluated with a staging PET scan. These patients are considered for downstaging and undergo LDLT only if good radiological response is documented or AFP drops below < 100 ng/ml. If not eligible for DS, patients with Vp1-2 MVI and low AFP are still considered for upfront LDLT.

The relatively small number of patients in the high-risk category (AFP > 1000 ng/ml or MVI) can be considered a limitation of the current study. We think it’s a substantial number as very few patients outside traditional transplant criteria, undergo LT with such tumor-related features (HCC 6.5–10 cm, any tumor number, MVI). The results of the current study are based exclusively upon LDLT experience and their application to DDLT is limited considering varying dynamics of prioritization and listing. Due to relatively small prognostic groups, a multivariate analysis to determine independent predictors of OS was not possible. Rates of LRT failure in our patients could not be reported since some patients were lost due to complex factors besides progressive liver failure and tumor burden. This, however, is not likely to have impacted outcomes in patients who eventually had LDLT for HCC.

The current study demonstrates acceptable post-transplant survival with more liberal expansion of cutoffs on tumor size and number with incorporation of AFP in the selection protocol. This approach makes it possible to consider LDLT even in selected patients with MVI. In the future, transplant criteria based on radiological and AFP response to LRT, and inclusion of biomarkers like AFP L-3 and PIVKAII might improve patient selection for LT.

## Supplementary Information

Below is the link to the electronic supplementary material.

Supplementary file1 (DOCX 12 KB)

## Data Availability

The data are available from authors upon reasonable request.

## Abbreviations

AFP: Alpha fetoprotein
LDLT: Living donor liver transplantation
LRT: Locoregional therapy
mRECIST: Modified response evaluation criteria in solid tumors
MVI: Macrovascular invasion
MWA: Microwave ablation
RFA: Radio frequency ablation
PEI: Percutaneous ethanol injection
PIVKAII: Prothrombin induced Vitamin K II antagonist
PVTT: Portal vein tumor thrombosis
DDLT: Deceased donor liver transplantation
RR: Recurrence risk
TACE: Trans arterial chemo embolization
UCSF: University of California San Francisco
