# Supplementary material for: Living donor liver transplantation for advanced hepatocellular carcinoma including macrovascular invasion
Source: J Cancer Res Clin Oncol. 2021 Jun 12;148(1):245–53. doi: 10.1007/s00432-021-03665-9 (PMC8752562; doi:10.1007/s00432-021-03665-9)
Supplement: Supplementary file 1 — Supplementary file1 (DOCX 12 KB) [file 432_2021_3665_MOESM1_ESM.docx]

**Supplementary table: Pattern of recurrence in patients with HCC who underwent LDLT**

| **Recurrence site** | **Number of recurrences (n=32)** |
| --- | --- |
|  | **Number (%)** |
|  | ***HCC without MVI (n=27)*** |
| **Lungs** | 15(55.6) |
| **Bones** | 10(37) |
| **Graft** | 6(22.3) |
| **Lymph nodes** | 6(22.3) |
|  | ***HCC with MVI(n=5)*** |
| **Lungs** | 4(80) |
| **Bones** | 2(40) |
| **Graft** | 2(40) |
| **Lymph nodes** | 1(20) |
